# Development of a Web-Based Resource to Support Driving Safety in Older Adults: Semistructured Interview and Focus Group Study

**DOI:** 10.2196/79630

**Published:** 2026-05-19

**Authors:** Mollie Cahill, Ranmalee Eramudugolla, Marwin Tang, Diane Hosking, Stephen Cratchley, Michael Regan, Lesley A Ross, Jasmine Price, Kaarin J Anstey

**Affiliations:** 1 School of Psychology, University of New South Wales, UNSW Sydney, High St, Kensington, Sydney, 2052, Australia, 61 02 9385 1000; 2 Neuroscience Research Australia, Sydney, Australia; 3 University of New South Wales, Ageing Futures Institute, Kensington, Sydney, Australia; 4 National Seniors Australia, Brisbane, Australia; 5 Suncorp Group, Brisbane, Australia; 6 Research Centre for Integrated Transport Innovation (rCITI), University of New South Wales, Sydney, Australia; 7 Department of Psychology, Clemson University, South Carolina, United States

**Keywords:** older drivers, ageing, driving safety, web-based platform, usability, fitness to drive, digital health, technology acceptance, digital literacy, co-design

## Abstract

**Background:**

Older adults face increased crash risk due to age-related declines in cognitive, visual, and physical functioning; yet, many Australians in their 70s are continuing to drive. Web-based platforms are increasingly used to deliver health and mobility information to older adults and may support safer driving; however, existing online resources on driving safety often lack age-specific guidance, have usability limitations, and may not be designed with older adults in mind.

**Objective:**

This study aimed to explore the information needs, preferences, and experiences of older drivers and general practitioners (GPs) to inform the formative, prelaunch development of a user-centered web-based platform, Ageing Well on the Road. Interviews and focus groups were conducted to gather feedback on content, accessibility, and usability, with data collection guided by the Consolidated Framework for Implementation Research and the Technology Acceptance Model frameworks to explore implementation and technology adoption factors.

**Methods:**

Website development was informed by a review of 26 Australian websites and consultations with stakeholders. Semistructured interviews were conducted with 7 older adults (aged >67 years; one additional participant completed only survey data) and a focus group with 10 GPs experienced in fitness to drive assessments for older drivers. Thematic analysis was used to explore participants’ experiences with online driving information and feedback on content, usability, and accessibility. Formative feedback from these users directly informed content and design refinements before public launch.

**Results:**

The website addressed priority topics, including age-related health conditions affecting driving, licensing requirements, vehicle safety features, and strategies for maintaining driving confidence. Older adults valued large fonts, plain language, intuitive navigation, and trustworthy, practical advice. GPs highlighted the website’s potential to support clinical discussions around driving capacity and safety. Barriers to engagement included digital literacy limitations, accessibility concerns, and uncertainty about online information credibility.

**Conclusions:**

A co-designed, web-based platform tailored to older adults and clinicians can enhance access to relevant, trustworthy information and support driving self-regulation. Involving end users in the development process is critical to optimize usability and ensure the resource aligns with their needs, highlighting the value of theory-informed, user-guided development for digital health resources.

## Introduction

Age-related declines in cognitive, sensory, and motor functions contribute to increased crash risk and driving difficulties among older adults [1,2]. Despite these challenges, over 90% of drivers in their 70s continue to rely on private vehicles, with many expected to drive well into their 80s [3]. Mobility plays a key role in preserving independence and well-being in late life, making it essential to support safe driving practices and provide accessible, evidence-based resources that help older adults make informed decisions about driving safety. However, many existing resources are not tailored to the unique needs of older adults, such as cognitive changes and digital literacy levels, highlighting a gap in accessible, targeted educational tools designed specifically for older drivers.

Web-based platforms have emerged as flexible and cost-effective methods for delivering health interventions, especially as over 75% of older adults now use the internet to seek health and mobility information [4]. While on-road driving assessments have shown effectiveness in evaluating driving ability [5,6], such services are not widely accessible. Older drivers, their family members, and clinicians often report difficulty locating clear, practical guidelines on driving safety, vehicle modifications, and licensing regulations [7,8]. While existing websites about driving safety provide general information, they frequently overlook the unique needs of older users, such as support with age-related decline, planning for driving retirement, and web design accessible to those with reduced physical or cognitive capacity [8].

Although digital health tools can broaden access to information, their effectiveness depends on how well they meet users’ expectations regarding usability, accessibility, and content relevance [9]. Older adults may face particular barriers to engaging with digital platforms, such as limited digital literacy, physical impairments, and uncertainty about the credibility of online information [10]. To address these challenges, theoretical frameworks can help guide the design and evaluation of new resources, as we first must understand how older adults search for information and what content they find useful. The Technology Acceptance Model (TAM), which has been applied in studies of older drivers’ adoption of advanced driver-assistance systems [11], suggests that perceived ease of use and perceived usefulness are key determinants of whether older adults engage with new technologies. This makes TAM a valuable lens for understanding how older adults evaluate online driving safety tools and identifying features that support or hinder uptake.

This study aimed to explore the information needs, preferences, and experiences of older drivers and general practitioners (GPs) with online driving safety content to inform the development of a user-centered web-based platform. A qualitative approach was used, including a review of existing online resources, consultations with stakeholders, and interviews with older adults (aged ≥67 years) and GPs. GPs play a key role in assessing older drivers’ medical fitness to drive (FtD) in Australia, yet often report uncertainty and a lack of appropriate tools to support these discussions [12,13]. These findings underscore the need for clear, accessible resources to support GPs in their decision-making. Given that internet use among older Australians aged >75 years continues to rise, with a sharp increase from 52% in 2019 (before COVID-19–related lockdowns) to 94% in 2022 [14], the development of a tailored web platform, Ageing Well on the Road [15], presents a timely opportunity to improve knowledge, support driving self-regulation, and enhance road safety. Findings from this study contribute formative insights to guide resource refinement and the development of best practice strategies for disseminating online driving safety tools targeted at older drivers.

## Methods

### Participants and Recruitment

Older adults (aged ≥67 years) were recruited to explore their information-seeking behaviors and perceptions of a newly developed web-based resource on driving safety. Recruitment was conducted via volunteer networks, social media, and partnerships with organizations such as National Seniors Australia. Additionally, participants from the Better Drive Trial and the Community in Health Aging Research Group who had previously consented to research participation were invited. GPs were recruited through the research team’s professional network and were eligible to participate if they were currently practicing and had experience conducting FtD assessments with older adults. Eligible participants received a link to an online screening and consent form hosted on REDCap (Research Electronic Data Capture; Vanderbilt University), a secure online survey platform. Those who consented were invited to participate in qualitative interviews or focus groups, where they were shown a prelive version of the website, allowing them to provide feedback on content, design, and usability before public launch, as well as to explore their broader information-seeking behaviors, preferences, and experiences with online driving safety content.

Eight older adults participated in the study, 7 of whom completed semistructured interviews, and 10 GPs participated in a single focus group discussion. Although the total sample size was modest (n=18), this is appropriate for the exploratory and qualitative nature of the study, which aimed to obtain rich, detailed accounts of participants’ perspectives rather than statistical generalizability [16]. In usability and user experience research, small samples are widely considered sufficient to identify the majority of issues, with previous studies showing that 5‐10 participants often reveal key concerns and that additional insights may be gained with up to 20 participants [17]. Recruitment continued until no additional expressions of interest were received, and the research team judged that sufficient feedback had been obtained to inform website development. As a pretesting stage, this sample provided practical insights into user experience, content clarity, and usability. Feedback from participants, together with input from the research team and stakeholders, directly informed iterative refinements to the website. Furthermore, the platform remains open to ongoing feedback via contact details provided on the site, allowing continued updates and improvements post launch.

### Website Development

A comprehensive review of 26 Australian websites addressing older driver safety was conducted by a research assistant under the supervision of the project team to assess existing resources, identify content gaps, and inform the development of a new web-based platform. Although not a formal systematic review, this process was guided by content experts, clinicians, stakeholder input, and team discussions, including a meeting with representatives from National Seniors Australia, the Australian Pensioners Insurance Agency (APIA), Suncorp Group, and Transport for NSW to ensure that relevant resources and key perspectives were captured. Websites were identified through targeted Google searches and recommendations from stakeholders.

The review highlighted several consistent patterns and gaps. Most government and national websites provided general information on licensing requirements, legal obligations for drivers with medical conditions, safe driving tips, and alternative transport options. Many included downloadable resources such as fact sheets, PDFs, and videos, and frequently linked to external resources rather than consolidating content in one place. However, few sites offered tailored guidance specifically for older drivers on maintaining driving skills, using vehicle technologies such as advanced driver assistance systems (ADASs), and supporting independence and decision-making around driving retirement. Accessibility features, interactive decision aids, and practical checklists were present in some resources, but not consistently across sites. Table S1 in Multimedia Appendix 1 provides a detailed overview of the content, format, and features of the 26 websites reviewed. Limited information on ADAS, such as lane-keeping assist, adaptive cruise control, and blind spot monitoring, was identified, despite evidence that these technologies can reduce crash risk and improve driving performance among older drivers, particularly when systems are acceptable and usable [18-20].

The resulting website, Ageing Well on the Road [15], was developed through an iterative, user-informed design process that incorporated feedback from older adults, GPs, and key stakeholders. The website design prioritized accessibility features appropriate for older adults, including clear headings, plain language, simple navigation structures, and large easy-to-read fonts. Stakeholder consultations involved representatives from an older adult advocacy group (National Seniors Australia), an older adult’s insurance agency (APIA), an insurance group (Suncorp Group), and a government transport authority (Transport for NSW), who emphasized the importance of trustworthy, practical resources that could be easily updated as regulations changed. Interview and focus group data directly informed the structure and content of the website. The resulting platform addressed content gaps identified in the review and reflected user and stakeholder input, with sections focused on maintaining driving skills with age, understanding and using ADAS technologies, planning for driving retirement, navigating licensing and medical requirements, and supporting family and carers. This approach aimed to produce an accessible, evidence-based resource to support safe driving and informed mobility decisions for older adults in Australia. A map of corresponding changes made to the website before launch is available in the Multimedia Appendix 1.

### Data Collection

Data were collected using an online questionnaire and through qualitative interviews and focus groups. The questionnaire captured consent, basic demographic information, and participants’ driving habits and attitudes toward licensing and safety. It included the participant information statement and consent form, which participants were required to review before proceeding. Consent was recorded electronically, with participants informed of their right to withdraw at any time. Interviews and focus groups were conducted via Microsoft Teams or Zoom (Zoom Communications) and ranged from 30 to 60 minutes in length. A semistructured interview approach was used, combining predetermined open-ended questions with flexibility for participants to elaborate on issues of relevance. The semistructured interview and focus group guides are provided in Multimedia Appendix 1.

Interview guides were informed by the Consolidated Framework for Implementation Research (CFIR), a widely used framework that provides a structured way of identifying the barriers and facilitators influencing the implementation and uptake of health interventions [21,22]. This framework allowed us to systematically design questions that explore contextual factors affecting the adoption of web-based driving safety resources. Specifically, questions about the credibility, usability, and perceived usefulness of the website reflected the domain of intervention characteristics; questions exploring licensing requirements, information sources, and available supports reflected the outer setting; and questions about older adults’ motivations, needs, confidence, and comfort with digital tools reflected individual characteristics. By aligning our guide with these domains, we ensured that data captured both personal experiences and broader implementation considerations, providing insights into factors that may support or hinder the uptake of health and safety interventions. This approach also allowed us to capture influences across multiple levels of context. For example, participants’ experiences with licensing regulations, access to transport, family involvement, and available community resources reflect the CFIR inner and outer setting domains, highlighting how both organizational and external factors shape engagement with driving safety interventions.

In addition, the TAM [11] guided the design of questions focused on digital engagement. TAM constructs of perceived usefulness and perceived ease of use shaped prompts asking participants to reflect on how straightforward the website was to navigate, whether the information was relevant to their needs, and whether they would recommend the resource to others. For clinicians, TAM principles were reflected in questions about preferred formats for digital screening tools and the likelihood of incorporating these into practice. These links allowed user feedback to be directly interpreted within established theoretical models of technology adoption, particularly for older populations. All sessions were audio-recorded, transcribed, and thematically analyzed. Feedback on user experience and content relevance was reviewed by the research team and stakeholders and directly informed website revisions to improve clarity, accessibility, and ease of use.

### Analysis

Thematic analysis was conducted by 2 researchers using NVivo (QSR International), following the phases described by Braun and Clarke [16,23]. The process involved familiarization with the transcripts, generating initial codes, allocating data segments to the codes, searching for themes, refining themes to ensure they worked in relation to the data, and developing the final thematic framework. Reflexive practices were incorporated throughout, including ongoing consideration of how researcher perspectives shaped coding and interpretation. Two researchers from different disciplinary backgrounds (paramedicine and psychology) independently conducted initial coding of the transcripts. Coding differences and emerging interpretations were discussed collaboratively, and themes were refined iteratively to incorporate multiple perspectives and support reflexivity, producing a more nuanced interpretation of the data [16,23].

Feedback on the website was analyzed primarily deductively, guided by the interview guide and research aim, which were informed by the CFIR and TAM. At the same time, themes related to participants’ broader knowledge needs, attitudes toward existing resources, and perceived barriers and facilitators were identified inductively from the data, so unexpected insights could be identified [24]. By combining framework-guided coding with open coding, we were able to use established theory to guide our analysis while also capturing new and unanticipated insights. The coding frameworks are provided in Multimedia Appendix 1.

Participant insights ultimately shaped the content, design, and functionality of the final website. Four main themes from the interviews and focus groups were identified: (1) barriers to accessing information and resources, (2) facilitators of online resource use, (3) driving safety and self-regulation, and (4) motivations and resource needs for information search. Themes were iteratively refined during analysis and further organized into subthemes at the reporting stage to aid clarity. Relevant formative feedback on the prelive website has also been incorporated to illustrate how these insights informed refinements to content, layout, and usability.

### Ethical Considerations

This study was approved by the Human Research Ethics Committee at the University of New South Wales (approval no iRECS6396), and participants provided written informed consent. No monetary compensation was provided to participants. Additionally, participants recruited from the Better Drive Trial were originally enrolled under separate approval from the University of New South Wales Human Research Ethics Committee (approval no HC190430) and had previously consented to be contacted for future research. All identifiable information was removed during data collection, and participants were assigned unique codes. The linking file connecting identities to study data was stored separately and was accessible only to the research team. Reidentification was not required, as no participants withdrew their data. Findings are reported in aggregate form to ensure that individual participants cannot be identified.

## Results

### Characteristics of the Older Adults

Eight older adults consented to participate via the REDCap survey, which collected basic demographics, driving habits, and interview availability. Seven participants completed the interviews. Participants’ ages ranged from 67 to 85 years. All were current drivers, with one participant reporting restrictions on their license, requiring spectacles and annual medical reviews. Most participants drove more than half the days of the week, with 3 driving 6 days per week and 3 driving 7 days per week. Weekly driving distances ranged from 31 km to over 150 km. When asked about how many more years they anticipated continuing to drive, the mean number was 17 (SD 6.84) years, with the range spanning from 8 to 30 years. Half of the participants reported having completed a medical review of their FtD by a doctor, while the other half had not.

Confidence in understanding licensing requirements after the age of 75 years was mixed. Three participants expressed high confidence, and 3 expressed low confidence, and the remaining participants reported neutral confidence. Confidence in managing age-related changes affecting driving safety was higher; 4 participants reported high confidence, and 3 reported moderate confidence. Most (n=6) participants also expressed high confidence in managing their own driving safety. Regarding satisfaction with existing information on licensing, aging, and driving safety, participants were generally neutral about both quality (n=4) and quantity (n=5). Common sources of accessing information included family members (n=5), doctors (n=4), and friends (n=2), with online resources being the preferred format for health and driving information (n=6).

### Characteristics of GPs

Ten GPs participated in a single focus group. Their ages varied, with 3 participants in their 30s, 2 in their 40s, 2 in their 50s, and 3 in their 60s. Three worked full-time and 7 part-time. Two had joined their practice within the past year, while the others had been with their practice for over 20 years. All GPs held a medical degree and Fellowship of the Royal Australian College of General Practitioners. Two also held PhDs, and 3 held other professional roles, such as being on the board of the Primary Health Network, postgraduate medical education, or running a GP training program.

### Barriers to Accessing Information and Resources

#### Reluctance to Acknowledge Driving Limitations

The older participant group identified a challenge in acknowledging the need for assessment, particularly among long-time drivers. One participant reflected on the difficulty of convincing long-time drivers to recognize when they may no longer be fit to drive, reflecting a broader hesitation in seeking out resources until necessary:

The biggest problem all around is persuading people who should be doing something more than an annual test… I think persuading people to use these things [online resources and tools]*,* is perhaps the key. And perhaps persuading someone who isn’t really aware of the difficulties they are having.

[P6, Male, 84]

This reluctance was echoed by the GPs, who noted that older adults typically do not recognize their own limitations when it comes to driving. One GP said:

It’s pretty rare… when a patient says “maybe I shouldn’t be driving.” I think that’s happened to me once. It may be family members that may say something, but it’s rare for the individual to say “no, I’m not good enough.”

[GP1]

These observations highlight the challenge both GPs and family members face and suggest the need for resources that help family members recognize signs of potential driving impairment and guide conversations about driving safety.

#### Reactive Information Seeking

Participants reported that they typically only sought information when it was urgently needed. As one older adult explained, “You’re not going to be prompted to go looking for information that you don’t need at the moment” (P3, Male, 67). This demonstrates a reactive approach to information-seeking, where older adults may not actively seek driving-related information unless prompted by personal circumstances or external cues such as medical diagnoses or legal requirements.

#### Skepticism Toward Online Resources

Concerns about the accuracy and reliability of digital tools emerged as another barrier. One older adult expressed doubt about online assessments: “I’m skeptical that a computer questionnaire can actually make an assessment on my ability to drive” (P4, Male, 77). This skepticism was combined with fears regarding privacy and potential misuse of personal data, indicating that trust in online resources remains a significant barrier for older adults, despite the widespread and increasing internet use and online screening tools.

#### Complexity of Licensing Regulations

Finally, participants highlighted that the complexity of state-by-state licensing regulations can cause confusion, especially for those who regularly travel across Australia. One older adult noted: “The laws are different in every state” (P7, Male, 71), pointing to the need for a centralized, comprehensive resource that consolidates licensing requirements and driving regulations across all states and territories into a single, accessible platform.

### Facilitators of Online Use

#### Accessibility and Convenience

Despite barriers, several factors were identified that could facilitate the use of online resources. Participants highlighted that web-based platforms are appealing because they are quick, easy, and convenient to use. One older adult explained: “It’s very quick. It’s very easy. It’s very comprehensive. You’ll get lots more answers” (P4, Male, 77). Similarly, another participant highlighted the convenience of online resources: “Well, it’s readily available and easy to access. I don’t have to go to the bookshop and look for it or look for a magazine” (P7, Male, 71). These comments underscore that the practical accessibility of online resources is a key facilitator for older adults.

#### Reducing Anxiety and Stigma

Online resources may also help alleviate the anxiety older adults feel about formal FtD assessments and the potential loss of their license. Participants in the GP focus group noted that many older adults experience significant stress around these assessments: “They get very anxious. Very worried they will fail and their license will be taken away from them, and there’s a rapid ball of catastrophising that goes on” (GP). This may discourage older adults from engaging in the process altogether, as one older adult described their personal reaction to medical appointments: “Every time I go to the GP, my blood pressure goes up, because outside the GP’s office my blood pressure is normal” (P5, Male, 85). While online resources cannot replace formal assessments, providing clear, transparent, and nonjudgmental information online may reduce the stress associated with seeking information from health care professionals and help individuals feel better prepared before attending in-person assessments. By framing these resources as supportive tools that clarify the process and expectations, they may reduce fear, facilitating greater acceptance and engagement with driving safety information.

#### Advisory Guidance and Reassurance

Some participants valued online tools that provided guidance rather than a definitive diagnosis, helping them feel more confident about their driving safety. One participant reflected on hesitating to use a potential dementia assessment tool, but noted a different approach for driving:

I discovered an online assessment for potential dementia, and I hesitated about doing it. I thought, maybe, I don’t want to know. I think that could be less the case in respect to driving. I would want to know, because I’d be putting other people at risk, other than just myself.

[P3, Male, 67]

This highlights how online tools can offer reassurance by helping individuals feel more confident that they are driving safely and not endangering others. Similarly, another participant emphasized the importance of feedback rather than prescriptive outcomes:

I wouldn’t mind as long as it’s a tool that’s helping you to determine if you’ve got some issues or not. But it’s not a definitive yes or no. You’ve got major issues, but something that sort of gives you feedback about how you answer the questions or deal with the online tools would be useful.

[P1, Male, 73]

Participants generally agreed that such tools should be “advisory and not prescriptive” (P2, Male, 74). These findings suggest that online resources can support older adults in managing their driving abilities in a nonthreatening and informative way, providing reassurance and reducing anxiety by framing driving safety as something manageable and shared rather than stigmatizing.

### Driving Safety and Self-Regulation

#### Proactive Approaches to Safety

Some participants took an active role in managing their driving safety by seeking assessments to maintain their driving confidence and reassurance. One participant described:

My specialist suggested I have this occupational therapy driving assessment, or something, which I did. I prepared for and did all the reading before that, so that’s basically how I got interested and realised that I can still drive properly and safely and confidently.

[P2, Male, 74]

This proactive approach to safety reflects how formal assessments can help older adults make informed decisions and continue driving with confidence, while also acknowledging the need for future adjustments as age-related declines (eg, reduced strength or flexibility) may affect certain driving-related skills, such as towing heavy vehicles.

#### Social Feedback and Family Influence

Social input was another critical component of driving self-regulation. Participants described relying on their peers or family members for feedback about their driving ability. As P4 (Male, 77) noted, “drivers will always talk to another driver about their ability or a problem.” Family concerns also influenced decisions, with one older driver recalling, “At one point, my wife’s mum, we were concerned about her driving, and we really didn’t want her to drive anymore. It’s one of those things—she was mid-80s at that stage” (P4, Male, 77). These examples show that self-regulation often occurs within a broader social context, influenced by both peer discussions and family involvement.

#### Self-Imposed Driving Restrictions

Self-regulation, including voluntarily limiting driving, was a frequent strategy to maintain safety, shaped by age-related caution or health concerns. For example, one participant explained:

My wife says that she isn’t going to drive after dark. She didn’t go and look and see something somewhere that said “this is how to know whether you can drive after dark or not.” She simply just said, “I don’t really feel that great driving after dark.”

[P6, Male, 84]

This self-imposed restriction was a common behavior among older adults, suggesting that the decision to stop driving may not always be based on formal assessments, but rather personal comfort and awareness of limitations. GPs confirmed this tendency, noting that many older adults consciously restricted their driving to familiar routes or comfortable distances. As one GP stated:

I find I have patients who self-restrict their own driving, and say, I only drive where I feel comfortable now…. they’ve just personally downgraded their driving status within their comfort level.

[GP1]

These findings suggest that self-regulation is a common and pragmatic response, and online tools could help validate these choices by providing reassurance and offering guidance about when to consult with a health care professional.

#### Access to Alternative Transport

The availability of public transport influenced decisions about driving cessation. Older driver participants living in urban areas reported that giving up driving was more manageable due to better access to amenities and public transport. As P6 (Male, 84) stated, *“*People in big cities (Melbourne, Sydney, etc) often give up [driving] quite easily as they are near the things they want and the public transport isn’t too bad.*”* In contrast, those in rural areas described more difficulty: “Country districts and places like Canberra… it’s going to be important and difficult to deal with. If you can’t get to the nearest shop” (P6, Male, 84). Limited transport alternatives in these areas make driving cessation more challenging, highlighting the need for tailored resources and support in this area.

### Motivations and Resource Needs for Information Search

#### Health-Related Prompts

Medical diagnoses were a key motivator for seeking information about driving requirements. For example, one older driver participant shared:

I’ve got mild cognitive impairment. And my specialist suggested I complete an occupational therapy assessment…, which I passed with flying colours. And now we’re continuing to drive safely and confidently, but it was a good wake-up call.

[P2, Male, 74]

Similarly, GPs noted that new diagnoses such as diabetes, syncope, or stroke often required them to notify licensing authorities, which in turn triggered patients to seek information about driving safety and regulatory requirements.

#### Family Influence and Forward Planning

Some participants were prompted to search for information because of concerns about their relatives, for example, researching licensing regulations after a relative’s dementia diagnosis. One older driver participant explained, “I looked online through the Roads and Traffic Authority, Roads and Maritime Services, and Service NSW websites, and just Googled generally about the issues” (P3, Male, 67). Such examples show how family experiences and concerns can indirectly motivate individuals to seek out resources and prepare for their own future driving needs. In contrast, some participants did not feel an immediate need for information, but acknowledged it would become relevant with age: “The legal requirements for drivers as we get to different ages would be a topic I’d certainly choose to Google” (P1, Male, 73). This reflects a more anticipatory approach, where individuals acknowledge that information will become useful as they age or if their circumstances change.

#### Preferred Sources of Information

Most participants preferred online resources for accessing driving-related information, with one older driver participant stating, “I prefer to use Google all the time” (P3, Male, 67), while others emphasized the need for these resources to be credible and relevant: “Well, I wouldn’t look at anything American for example, so relevant to my location. But not put there by an insurance company, it’s got to be a reputable source” (P7, Male, 71). This emphasis on trustworthiness shows the importance of ensuring that online resources are not only easy to access but also authoritative and specific in their content.

#### External Prompts and Regulatory Triggers

Despite varied preferences, most participants reported that information-seeking often occurred reactively, in response to external prompts. These included medical advice, legal obligations, family member suggestions, or official reminders. As one GP described:

Someone newly diagnosed as a diabetic has to be notified, or a syncope episode or a stroke—those sorts of things—I’ll sometimes go and notify the RMS [Roads and Maritime Services] for them, as at least you know that way it’s done.

[GP3]

This illustrates how medical diagnoses such as diabetes or syncope can motivate both health care professionals and patients to seek relevant driving safety information and comply with legal requirements. Similarly, one older adult explained, “I got a letter in the mail saying you are due for a check-up… It’s a push technique, not a pull” (P6, Male, 84). These examples illustrate how external systems, in addition to personal initiatives, often drive engagement with information about licensing and driving safety.

#### Preliminary Impressions of the Ageing Well on the Road Website

Participants were invited to review a prerelease version of the Ageing Well on the Road website and provide formative feedback. This input guided refinements to content and design before public launch, as a full usability evaluation was beyond the scope of this study. Older adults described the site as clear, accessible, and easy to navigate. One participant noted: “It wasn’t too detailed, and it was in plain English…. I thought it was easy to read, and well presented” (P1, Male, 73), while another commented: “I thought it was easy to navigate, it was broken up into convenient tiles… it included things like technically advanced motor vehicles, and getting cars to assist with lane control etc” (P4, Male, 77). Credibility associated with the university affiliation was also highlighted as supporting trust in the information: “It’s got the University of New South Wales symbol... so that gives it some credibility” (P7, Male, 71).

Specific content elements were particularly valued, including state-based licensing information and visual resources. One participant explained: “One aspect I found very good was that you had each state and territory listed, and their licensing requirements” (P4, Male, 77), while another added: “There was a graphic about the blind spots for heavy vehicles, and that was informative to me, because I didn’t realize just how blind they are” (P3, Male, 67). Participants indicated they would likely recommend the website to peers, families, and community groups once it is live, noting, “Once it’s online, then I’ll certainly be promoting it as a good source of information” (P3, Male, 67). Overall, feedback was positive, and participants’ broader insights on information needs, preferences, and driving safety concerns were used alongside this input to refine the website before public launch.

GPs also provided positive feedback and highlighted the site’s potential use in clinical practice. One GP commented that the site “looks great” and that they would “absolutely refer patients to this.” Suggested refinements included adding practical financial guidance for patients retiring from driving, such as demonstrating potential savings from selling a vehicle vs ongoing taxi or ride-share costs, and including information on taxi subsidy schemes. GPs recommended incorporating research-based statistics and evidence on driving frequency and skill decline to support clinical discussions. These suggestions informed adjustments to content and resources before the public launch.

## Discussion

### Principal Findings

This work represents formative, prelaunch development of the website and aimed to explore how older adults seek information about driving requirements and safety, their motivations and resource needs, and how GPs support older adults in making FtD decisions. Insights on online health-seeking behaviors, information preferences, and driving-related concerns informed the development of the website. Our findings suggest that older adults’ information-seeking behaviors are often needs-driven, influenced by personal circumstances, medical conditions, and external prompts, such as family concerns or government notifications, as identified in interviews with older adults and GPs. This aligns with prior research suggesting that older adults often seek information when prompted by a specific need or external factor [25,26].

A key observation was that individuals with existing medical conditions, such as mild cognitive impairment, were more likely to actively seek information. For example, one participant described how a specialist’s recommendation for an occupational therapy assessment prompted his search, reinforcing the belief that health-related triggers often lead to increased engagement with driving-related information [3]. Similarly, those with familial experiences of cognitive decline, such as dementia, sought information to better understand the legal and safety implications of continued driving. This finding mirrors studies that emphasize the important role family members and caregivers play in initiating conversations about driving cessation [27]. In contrast, participants who had not yet faced challenges to their driving ability expressed little motivation to seek information. This supports the idea that information-seeking behavior among older adults is often “needs-based” and typically occurs when it becomes personally relevant [26]. However, participants noted that legal requirements would become important over time, indicating they were aware of the information’s future relevance even if not immediately needed.

Some participants in the interviews mentioned holding specialized driving licenses, such as for heavy vehicles or trucks. Unsurprisingly, these participants were generally more familiar with regulatory requirements due to the mandatory annual medical assessments these licenses require. These individuals did not actively seek out additional information, as they felt their licensing processes inherently provided them with updates on medical and cognitive evaluations. This supports the idea that external regulatory frameworks can be effective in disseminating information to older adults, as also found in previous studies focused on policy-driven health behaviors [28].

Regarding preferred information sources, most participants favored online searches, especially Google and government websites. This suggests a need for user-friendly, government-verified online resources tailored to older adults, reflecting other findings from studies that show older adults are increasingly turning to digital platforms for information [29]. Building on these findings, research suggests that internet use can improve the physical and mental health of older adults [30] and that digital literacy is a key factor influencing engagement with online health information. Older adults can be disadvantaged in terms of physiological function, cognitive ability, social status, and economic status, which can limit their use of electronic technology compared with younger individuals [30]. Those with higher digital literacy are more confident navigating websites, evaluating content credibility, and applying information in daily life, whereas those with lower digital literacy may encounter barriers to effectively using digital resources [30,31]. Considering digital literacy and the local sociocultural context when designing a web-based platform could enhance accessibility, usability, and overall engagement, ensuring that the online resource is inclusive for older adults with varying levels of technological skill. When interpreted through the lens of CFIR and TAM, several constructs were particularly relevant in shaping user perceptions. For CFIR, intervention characteristics (such as perceived credibility and usability of the website) and individual characteristics (including confidence and comfort with digital tools) strongly influenced engagement. For TAM, perceived usefulness and perceived ease of use were key determinants of how participants evaluated and were willing to adopt new digital resources [11].

A key theme that emerged was the prevalence of a “push” rather than “pull” approach to information seeking. Many participants reported they did not seek out information unless prompted by an external cue, such as a family intervention or a government letter advising them to go for a medical check-up. This aligns with behavioral models of health communication, which suggest that external cues are often the primary motivators for action [32]. Overall, while some adults proactively sought driving-related information, many reported that they rely on external triggers. To promote safe driving behaviors among aging populations, efforts should leverage these “push” mechanisms to ensure older drivers remain informed and make safe decisions while driving.

In addition to the individual benefits, improving driving safety in older adults has broader societal implications. Older drivers are often overrepresented in crash and injury statistics [11], so supporting safer driving practices through proactive self-regulation and education in later life can contribute to the overall safety of all road users. Therefore, derisking older driver behavior is not only important for maintaining independence and mobility, but also for potentially reducing the public health burden associated with road traffic incidents.

Feedback on the prelive website was constructive and informed content and presentation refinements, providing an initial indication of reach and potential usability. Future research should be conducted to examine longer-term use, gather structured user feedback, and investigate how digital resources can be optimized for this demographic, including strategies to encourage proactive information-seeking. The website does not collect any user data, but visitor metrics, such as the number of views, are monitored monthly to provide ongoing insights into user engagement.

### Limitations

This study involved a small, qualitative sample that offers in-depth insights, but limits generalizability to the broader population. The older adult sample was predominantly male and recruited from participants already engaged with research projects, potentially introducing positive bias. Limited demographic information was available for the GP focus group, as the hosting practice did not collect full participant-level data to minimize burden, with only summary information on age ranges, work status, and qualifications available, and sex was not reported. Study findings reflect the Australian licensing and health context and may differ in other jurisdictions. This work was formative and undertaken before the website’s public launch. While participants’ perspectives informed the website design, this study was not a formal usability evaluation. A comprehensive postlaunch evaluation was outside the scope of this project and will require further funding, but would be beneficial, with more diverse users and regions.

### Conclusions

This study provides insight into factors influencing older adults’ engagement with driving safety resources. Findings suggest that information-seeking is often reactive, prompted by health issues, family concerns, or legal requirements. Barriers to engagement include skepticism about online resources, the complexity of licensing regulations, and reluctance to acknowledge the need for driving assessments, highlighting the importance of accessible, trustworthy, and user-friendly resources. External cues, such as medical recommendations and family and caregiver input, can encourage engagement with driving safety resources. GPs play a critical role, often serving as the first point of contact for older adults experiencing health issues affecting their driving. Proactive GP involvement, coupled with clear, reliable digital resources, may support older adults in seeking and applying relevant information. Future research should explore how health care professionals, particularly GPs, can be better supported and educated to enhance their role in FtD assessments.

Feedback from older adults, GPs, and stakeholders informed the formative, prelaunch development of the Ageing Well on the Road website [15]. Insights guided the creation of a dedicated clinician section and shaped the site’s plain-language content, intuitive navigation, and additional resources for family members and caregivers, who often play a critical role in initiating conversations about driving safety and helping older adults navigate relevant information. The multiuser design aims to increase accessibility and promote shared decision-making. Stakeholders involved in the co-design process included representatives from National Seniors Australia, APIA, Suncorp Group, and Transport for NSW, reinforcing the need for accurate, trustworthy, and up-to-date content. Together, these contributions helped create a user-informed, accessible web-based resource tailored to the real-world needs of older adults and clinicians.

Overall, this formative work indicates that a co-designed, web-based platform has the potential to simplify access to essential information, build trust in digital content, and encourage early engagement by older adults and GPs. By addressing key barriers, the platform may contribute to safer driving practices, support independence, and inform broader road safety initiatives. Although developed for an Australian context, the platform’s structure could be adapted internationally, with state- or region-specific driving regulations mapped to local legal requirements while maintaining the multiuser design and content approach.

## Supplementary material

10.2196/79630 Multimedia Appendix 1 Website review, methodological details, and coding framework.

## Abbreviations

ADAS: advanced driver assistance system
APIA: Australian Pensioners Insurance Agency
CFIR: Consolidated Framework for Implementation Research
FtD: fitness to drive
GP: general practitioner
REDCap: Research Electronic Data Capture
TAM: Technology Acceptance Model

## References

[1] Anstey KJ, Eramudugolla R, Ross LA, Lautenschlager NT, Wood J (2016). Road safety in an aging population: risk factors, assessment, interventions, and future directions. Int Psychogeriatr.

[2] Toups R, Chirles TJ, Ehsani JP (2022). Driving performance in older adults: current measures, findings, and implications for roadway safety. Innov Aging.

[3] Anstey KJ, Li X, Hosking DE, Eramudugolla R (2017). The epidemiology of driving in later life: sociodemographic, health and functional characteristics, predictors of incident cessation, and driving expectations. Accid Anal Prev.

[4] Tennant B, Stellefson M, Dodd V (2015). eHealth literacy and Web 2.0 health information seeking behaviors among baby boomers and older adults. J Med Internet Res.

[5] Anstey KJ, Eramudugolla R, Kiely KM, Price J (2018). Effect of tailored on-road driving lessons on driving safety in older adults: a randomised controlled trial. Accid Anal Prev.

[6] Castellucci HI, Bravo G, Arezes PM, Lavallière M (2020). Are interventions effective at improving driving in older drivers?: A systematic review. BMC Geriatr.

[7] Betz ME, Haukoos JS, Schwartz R (2018). Prospective validation of a screening tool to identify older adults in need of a driving evaluation. J Am Geriatr Soc.

[8] Levasseur M, Coallier JC, Gabaude C (2016). Facilitators, barriers and needs in the use of adaptive driving strategies to enhance older drivers’ mobility: importance of openness, perceptions, knowledge and support. Transportation Research Part F: Traffic Psychology and Behaviour.

[9] Lackie ME, Parrilla JS, Lavery BM (2021). Digital health needs of women with postpartum depression: focus group study. J Med Internet Res.

[10] Radovic A, Odenthal K, Flores AT, Miller E, Stein BD (2020). Prescribing technology to increase uptake of depression treatment in primary care: a pre-implementation focus group study of SOVA (Supporting Our Valued Adolescents). J Clin Psychol Med Settings.

[11] Hansen A, Kiely K, Attuquayefio T (2025). Assessment of the application of technology acceptance measures to older drivers’ acceptance of advanced driver-assistance systems. Appl Ergon.

[12] Jones K, Rouse-Watson S, Beveridge A, Sims J, Schattner P (2012). Fitness to drive - GP perspectives of assessing older and functionally impaired patients. Aust Fam Physician.

[13] Stefanidis KB, Summers MJ, Clark M (2025). The assessment of cognitive function and medical fitness to drive in older adults: a qualitative study with general practitioners. J Transp Health.

[14] (2024). Communications and media in Australia series: how we use the internet executive summary and key findings. Australian Communications and Media Authority.

[15] (2024). Ageing well on the road. Driving, Ageing and Health Research Team.

[16] Braun V, Clarke V (2019). Reflecting on reflexive thematic analysis. Qual Res Sport Exerc Health.

[17] Faulkner L (2003). Beyond the five-user assumption: benefits of increased sample sizes in usability testing. Behav Res Methods Instrum Comput.

[18] Masello L, Castignani G, Sheehan B, Murphy F, McDonnell K (2022). On the road safety benefits of advanced driver assistance systems in different driving contexts. Transportation Research Interdisciplinary Perspectives.

[19] Young KL, Regan MA, Triggs TJ, Tomasevic N, Stephan K, Mitsopoulos E (2007). Impact on car driving performance of a following distance warning system: findings from the Australian Transport Accident Commission SafeCar project. J Intell Transp Syst.

[20] Regan M, Stephan K, Mitsopoulos E (2007). The effect on driver workload, attitudes and acceptability of in-vehicle intelligent transport systems. J Road Safety.

[21] Damschroder LJ, Reardon CM, Widerquist MAO, Lowery J (2022). The updated Consolidated Framework for Implementation Research based on user feedback. Implement Sci.

[22] O’Connor CM, Poulos CJ, Kurrle S, Anstey KJ (2023). Bridging the gap: study protocol for development of an implementation strategy for evidence-informed reablement and rehabilitation for community-dwelling people with dementia. Arch Gerontol Geriatr.

[23] Braun V, Clarke V (2006). Using thematic analysis in psychology. Qual Res Psychol.

[24] Sharp P, Bottorff JL, Rice S (2022). “People say men don’t talk, well that’s bullshit”: A focus group study exploring challenges and opportunities for men’s mental health promotion. PLoS ONE.

[25] Fang Z, Liu Y, Peng B (2024). Empowering older adults: bridging the digital divide in online health information seeking. Humanit Soc Sci Commun.

[26] Shen X, Helion C, Smith DV, Murty VP (2024). Motivation as a lens for understanding information-seeking behaviors. J Cogn Neurosci.

[27] Adler G, Rottunda S (2006). Older adults’ perspectives on driving cessation. J Aging Stud.

[28] Hakamies-Blomqvist L, Henriksson P (1999). Cohort effects in older drivers’ accident type distribution: are older drivers as old as they used to be?. Transp Res Part F Traffic Psychol Behav.

[29] Zhao YC, Zhao M, Song S (2022). Online health information seeking behaviors among older adults: systematic scoping review. J Med Internet Res.

[30] Shi Z, Du X, Li J, Hou R, Sun J, Marohabutr T (2024). Factors influencing digital health literacy among older adults: a scoping review. Front Public Health.

[31] Yang Y, Yao X, Lu D (2024). Improving the eHealth literacy of older adults: a scoping review. Geriatr Nurs (Lond).

[32] Weinstein ND (1988). The precaution adoption process. Health Psychol.

