# Supplementary material for: Development of a Web-Based Resource to Support Driving Safety in Older Adults: Semistructured Interview and Focus Group Study
Source: JMIR Aging. 2026 May 19;9:e79630. doi: 10.2196/79630 (PMC13186436; doi:10.2196/79630)
Supplement: Multimedia Appendix 1 [file aging-v9-e79630-s001.docx]

| Website name | Provider type | URL | State | Main topic | Content | Format | Potential features for website |
| --- | --- | --- | --- | --- | --- | --- | --- |
| NSW Government - Driving, Boating and Transport | Government | https://www.nsw.gov.au/driving-boating-and-transport/driver-and-rider-licences/older-drivers-and-riders | NSW | Driver and rider licensing for older adults (70+) | - Licence renewal and modification processes for older drivers - Assessments required by licence type and age - Expectations for assessments - Guidance on retiring from driving - Information for friends and family concerned about an older adult’s driving - Related health and disability conditions (e.g., eyesight, dementia) | - Accessibility feature (ReadSpeaker webReader) - Printable PDFs (e.g., "A Guide to Older Driver Licensing") - Shareable via social media (Facebook, LinkedIn, Twitter, email) - Hyperlinks to related topics - Checklist for driver readiness - Search tool for locating older driver assessors | - Content on retiring from driving - List and locator tool for older driver assessors/instructors - Accessibility enhancements (e.g., ReadSpeaker webReader) - Central linking to all state older driver handbooks |
| NSW Government – On the road 65Plus | Government | https://www.transport.nsw.gov.au/roadsafety/older-road-users | NSW | Road safety and mobility for older adults (65+) | - Overview of aging-related conditions affecting driving (vision, hearing, memory, flexibility, medications) - Dementia and driving: impacts, signs, and legal requirements - Signs of declining driving ability - Safe driving tips (before and during trips) - Guidance on choosing a safe vehicle - Alternative transport options (walking, public transport, taxis, etc.) - Links to Centre for Road Safety resources and road rule videos | - Clickable headings for easy navigation - Expandable pop-down boxes - Hyperlinks throughout - Decision aids (from University of Wollongong) - Handbooks available in 11 languages - Video animations of road rules - Statistics and links to external organisations | - Information on recognising signs of declining ability - Safe driving advice embedded throughout - Practical retirement-from-driving advice and transport alternatives - Hover-over expansions for keywords - Use of video animations to explain complex rules |
| Access Canberra – ACT driver licence information | Government | https://www.accesscanberra.act.gov.au/driving-transport-and-parking/licences | ACT | Driver licensing requirements and medical conditions for ACT residents | - Brief section on older driver licence requirements - Responsibilities of drivers with medical conditions (e.g., cognitive impairment) - Overview of occupational therapy (OT) driving assessments and what they evaluate (cognition, motor function, reaction time, road rules) - Information on licence conditions, suspensions, and cancellations - Details on medical assessments - Alternative transport resources | - Hyperlinks to forms, internal pages, and external resources | - Clarifies responsibility for self-reporting or reporting others unfit to drive - Explains the purpose and components of driving assessments - Describes conditional licences as an option for continued, restricted driving |
| ACT Older Drivers Handbook | Government – Council on the Ageing (COTA) | https://files.accesscanberra.act.gov.au/legacy/4715/ACT%20Older%20Drivers%20Handbook.pdf | ACT | Driving, ageing, health, and independence for adults aged 65+ | - Promotes safe mobility choices (driving, walking, public transport) - Age-related effects on cognition, sensory and motor skills - Health conditions that affect driving (e.g. diabetes, eyesight, heart disease) - Licensing and driving assessments in ACT - Dementia and driving - Alcohol and medication impacts - Choosing and maintaining vehicles with ADAS - Driving in challenging conditions - Post-crash steps - Life beyond driving and transport alternatives - Support for concerned family/friends | - Colourful, accessible PDF handbook (online or print) - Checklists, illustrations, content page, references - External links and contact info - Less formal and more user-friendly than other government resources | - Covers non-driver mobility (pedestrian/public transport tips) - Guidance for driving with specific conditions - Practical driving tips for challenging scenarios - Steps to take post-collision - List of relevant organisations for support - Medication impacts explained clearly |
| Seniors Road Safety – NT | Government | https://roadsafety.nt.gov.au/safety-topics/seniors | NT | Promoting road safety for seniors within broader NT road safety strategy | - NT licensing requirements and assessment process for older drivers - Mobility scooter regulations - Tips for reducing road deaths and injuries among seniors - Road rules, penalties, and responsibilities - Some content broadly focused (e.g., drugs/alcohol, roadworks, passenger safety) and not specific to older drivers | - Range of downloadable resources: brochures, handbooks, fact sheets, posters (some in multiple languages) - Activity sheets, FAQs - Videos, animations, and audio clips - Crash statistics (NT-specific and national) - External links to additional resources | - NT-specific crash statistics and comparisons with national data - Public awareness around penalties and legal consequences - Emphasis on not only crash prevention but also reducing harm from crashes - Opportunity to tailor resources more directly to older adults (currently more general) |
| Towards Zero Together – Older Drivers | Government | https://towardszerotogether.sa.gov.au/safe_road_users/older_drivers | SA | Safer people, vehicles, speeds, and roads, with limited focus on older drivers | - Limited direct content on ageing and driving - Information on how medications affect driving - Brief content on elderly pedestrian safety - Links to the “Moving Right Along” booklet covering: driving safely for longer, medical conditions and medications, legal obligations, driver assessments, and reducing or giving up driving | - "Moving Right Along" booklet for older drivers and their support network - Fact sheet with older road user stats - Email subscription option - News section (last updated mid-2022) - Hyperlinks to other publications and resources | - Information on how common medical conditions (beyond medication use) can affect driving - Option to receive updates on new content via email - Highlighting and promoting the “Moving Right Along” booklet as a central resource - Opportunity to expand and update news and research sections more regularly |
| My Licence – Safe Driving Tips for Older Drivers | Government | https://www.mylicence.sa.gov.au/safe-driving-tips/older-drivers | SA | Driving safety, licensing, and older driver responsibilities | - Safe driving tips tailored for older adults - Responsibilities related to fitness to drive - Relevant licensing and medical assessments - List of health conditions affecting driving - Retirement from driving and alternative transport - Information for concerned family/friends - Link to "Moving Right Along" handbook | - Links to social media - Current safety campaign banners - Interactive quizzes - Handbooks - Conversation planner for discussing driving concerns - Short videos (Dementia and Driving Project) - PDFs and external resource links | - Banners for latest updates or resources - Interactive conversation tools for family/friends - Embedded videos supporting decision-making (e.g. Dementia and Driving Project) - Strong support for third parties helping older drivers |
| On The Right Track – Older Drivers | Government | https://www.dit.sa.gov.au/ontherighttrack/driving_safely/look_after_yourself/older_drivers | SA | Aboriginal road safety and older driver licensing | - Guidance for older individuals who feel they may no longer be safe to drive - Links to other SA resources - Licensing and driver responsibilities - Safe driving advice: medications, fatigue, stress - Respect for other road users - Basic vehicle maintenance - Driving conditions (e.g., poor weather, overtaking) - Crash and emergency response steps | - Visual elements including Aboriginal artwork and avatars - Click-through driving tips - Downloadable PDFs - Links to external websites - Contact form for cultural representation - Email subscription - Search bar | - Practical advice on managing stress and fatigue while driving - Emphasis on how unsafe driving affects others - Step-by-step response for vehicle issues (e.g., brake failure) - Interactive, culturally sensitive design and language inclusion options |
| Transport Accident Commission – Older People | Government | https://www.tac.vic.gov.au/road-safety/road-users/older-people | VIC | Road safety for older road users (drivers and pedestrians) | - Signs that an older person’s driving may be declining - Safe driving tips for older drivers - Pedestrian safety advice - Information on stopping driving - How medications affect driving ability - Table of medications that impair driving, with usage, type, and names - Guidance for those concerned about an older person’s driving | - Icons and images - Facts and checklists - Tables and brochures - Available in multiple languages - Links to related content - Contact info and subscription option | - Clearly outlined signs of driving decline and what to do - Table-format medication information: type, name, purpose, and impact - Multi-language support for accessibility - Printable resources for clinicians and family members |
| VicRoads – Health and driving | Government | https://transport.vic.gov.au/registration-and-licensing/licences/medical-conditions-and-reviews/how-medical-reviews-work | VIC | Health, ageing, and driving safely | - Ageing and how it affects driving - Responsibilities of older drivers - Tips for reducing risk - Choosing a safe car and modifying it - Driving with medical conditions or disabilities - Reporting health conditions and unsafe driving - Effects of specific conditions ( e.g., dementia, heart disease) - Monitoring health (sleep, fitness, alcohol, etc.) - Vision and driving - Disability and licensing - Vehicle modifications - Stopping driving: signs, assessments, medical reviews | - Ageing and safe driving handbook (includes a self-assessment guide) - Fact sheets and handbooks (some in other languages) - Videos (e.g., on visual impairments) - Tables and checklists - Contact and support info - Personal stories - Internal/external links - FAQs and enquiry form | - Tools for self-monitoring (e.g., checklists) - Health-focused driving safety education (sleep, fitness, alcohol) - Personal stories and quotes from older drivers - Videos visualising impairments - Easy access to VicRoads for support via message box |
| Victoria Police – Older drivers | Government | https://www.police.vic.gov.au/older-drivers | VIC | Staying safe on the road as an older driver | - Signs of declining road safety that someone over the age of 65 may have - Ways to reduce the chance of an accident and to keep driving for longer - Fact sheets on older pedestrians, car parks, fitness to drive, distraction, and retiring from driving | - Lists/checklists - Quarterly articles/fact sheets downloadable as PDFs | - Specific information on navigating common scenarios like car parks or driving with passengers - Regularly released articles/fact sheets for seniors |
| Government of Western Australia – Road Safety Commission - Seniors | Government | https://www.wa.gov.au/organisation/road-safety-commission/seniors | WA | The voice of road safety in WA with the aim to decrease road trauma | - Why road safety decreases as one ages (medication, vision, and flexibility) - Licensing requirements for seniors - Alternative transportation - Road rules - General information on dangerous driving behaviours and avoiding them | - Recently updated - Recent announcements and news - Streetwise: scenarios + quizzes - Statistics - Posters, fact sheets, resources - Tables - Social media links - Community Connect portal - External links within the website | - Displaying road scenarios via video/images with rule explanations and penalties (quiz-style) - Social media integration - Online forum for seniors and road safety |
| Queensland Government - Seniors | Government | https://www.qld.gov.au/seniors | QLD | Content relating to senior Queenslanders | - Requirements for drivers over the age of 75 (particularly regarding medical certificates) - Notification of medical conditions - How ageing affects driving – vision, movement, and information processing, with some driving safety tips - Brief two sentences on car modifications for older drivers - Surrendering your licence - General info on driving safely (e.g., weather, country roads, trucks) - Support services for those who have lost driving independence | - Search bar - Printable - Links to resources (internal and external) - Updated mid-2022 - Lists - Tables (e.g., driver rest areas) - Downloadable PDFs | - Car modifications tailored for older drivers with changing physical/cognitive needs - Easy-print icon/button - Maps/tables showing rest areas and travel services |
| Tasmanian Government Transport Services – Health and driving | Government | https://www.transport.tas.gov.au/licensing/health_and_driving | TAS | Health (medical conditions, disabilities, treatments, and ageing) and the effect on driving and licensing | - Responsibilities with driving as you age - Tasmanian Older Drivers Handbook – licensing, self-evaluating driving ability, medical conditions and driving, retiring from driving - Fitness to drive and legal responsibilities to report medical conditions/undertake assessments - What medical conditions affect driving – how they affect it, symptoms, and precautions - What to do if concerned about someone else's driving - Vehicle modifications for disabilities | - FAQs in a pop-down box format - Key links to documents (PDF/Word) and internal webpages - Handbooks and brochures | - Frequently asked questions that expand on click to reveal answers (accordion format) |
| Austroads – Assessing Fitness to Drive | National body (Austroads & National Transport Commission) / Sixth edition 2022 | https://austroads.gov.au/drivers-and-vehicles/assessing-fitness-to-drive | National | National driver medical standards | - Geared toward health professionals, but includes driver information - Medical conditions that affect driving - Driver responsibilities regarding medical conditions - Age-related changes and driving management - Licensing, fitness to drive, and health professional assessments | - Online, PDF, and Word versions - Search bar - FAQs - News section (last updated 2022) - Email subscription - Internal/external links (including state/territory resources) - Fact sheets | - Information for health professionals about assessing older drivers - What older drivers should expect when being assessed by a GP or other health professional |
| RoadSafe Westgate Community Road Safety Council | Community-based; info sourced from Vic Police, VicRoads, instructors | https://roadsafewestgate.org.au/older-road-users-safety/ | VIC | Keeping older drivers on the road longer while safe | - Information on presentations/events relevant to the Westgate community - Risks of being an older driver, but also highlights the positives | - Pictures (stock and community images) - Calendar for latest posts - Enquiry form | - Emphasis on the positives/good qualities of older drivers - Calendar feature for news or events |
| ADHERe | Developed with input from health professionals, older drivers, families, transport experts. Review planned in 2026 | https://adhere.org.au/olderdrivers/ | NSW | Aged Dementia Health Education and Research | - A decision aid for deciding whether to continue, reduce, or stop driving - Tips for driving safely longer or modifying driving – Licensing requirements - Alternative transport options - Vehicle safety/driving risks - Skills needed for driving and warning signs of decline - Medical conditions that impact driving | - Videos - Interactive decision aid (online, PDF, printable) - External links - Quotes/stories from older drivers - Checklists/lists - Quizzes - Customizable worksheets with downloadable responses - Stock images - Statistics | - Behavioural tips for managing declining driving ability (e.g., avoid night driving) - Self-assessment tools or worksheets that allow users to input and download their answers |
| COTA | Varies depending on the State/Territory; Mainly sourced from government websites | https://cota.org.au/ | National | Promoting the rights and interests of older Australians | - Scenarios illustrating when driving safety may decline - Tips for maintaining safe driving and driving within capability - Living without a licence and maintaining independence - Road rules, including navigating intersections and road scenarios/conditions - Vehicle checks | - Search bar - Each state/territory has its own specific website - Newsletters - Checklists - Media releases and submissions to inquiries - Comment sections on some pages - Links to relevant external websites - Brochures - Pictures/diagrams - Handbooks (e.g., Guide to Driving on WA Roads) | - Use of scenarios to demonstrate what declining driving ability looks like  -Separate website for each state/territory |
| National Seniors | Possibly sourced from studies, universities, research institutes, and government websites; some not sourced at all | https://nationalseniors.com.au/ | National | NFP for Australians over 50 | - Driving safety - How ageing affects driving - ADAS (from the UNSW-NSA report) - Car maintenance and vehicle safety - Provides roadside assistance | - Search bar - Links to social media pages - Shareable news articles - Statistics - Fact sheets - Newsletters - Links to external resources and websites | - Information on roadside assistance or other services for drivers experiencing car trouble |
| Dementia Australia | Direct information from people with dementia, carers, and dementia service providers; government website; other organisations like NRMA or Alzheimer’s Australia NSW | https://www.dementia.org.au/living-dementia/staying-connected/driving-and-dementia | National | Dementia and driving | - Targeted towards individuals with dementia, but also towards family members - Also has separate pages for each state and territory - Licensing requirements - Signs that dementia is affecting someone’s driving - Conversations regarding safety concerns and retiring from driving - Driving alternatives | - Targeted towards individuals with dementia, but also towards family members - Also has separate pages for each state and territory - Licensing requirements - Signs that dementia is affecting someone’s driving - Conversations regarding safety concerns and retiring from driving - Driving alternatives | - Targeted towards individuals with dementia, but also towards family members - Also has separate pages for each state and territory - Licensing requirements - Signs that dementia is affecting someone’s driving - Conversations regarding safety concerns and retiring from driving - Driving alternatives |
| Apia Good Life | Government websites; some information not sourced; some inputs from experts e.g., an exercise physiologist | https://www.apia.com.au/apia-good-life.html | National | Insurance for over 50s; Good Life provides articles and offers | - Information on driving safety in the form of articles - How ageing can affect driving (hearing, vision, response times) - Signs that an older person may be unsafe on the road - How to ensure your older family member/friend is driving safely - How to maintain health to ensure safety on the road - Legal responsibilities (assessments, reviews, certificates) when driving over a certain age by state | - Quarterly newsletter - Search bar - PDF guides - News articles, sometimes with inputs from experts e.g. an exercise physiologist - Internal and external links | - Provide information with inputs and quotes directly from experts |
| NRMA | Information from government, input from other organisations, NRMA surveys and reports, some information unreferenced | https://www.mynrma.com.au/ | NSW and ACT | Advocacy for road users | - Driving assessments and senior driver training - Licence requirements - General road rules, distracted driving, not specifically targeting seniors | - Brochures  - Search bar  - Social media links | - Information on the purpose and process of driving assessments, including evaluation criteria - Guidance on finding driving programs, lessons, assessors, and training options |
| Budget Direct | Government, news articles, and websites. Disclaimer that sources may be inaccurate/outdated; articles are not dated and likely not updated. | https://www.budgetdirect.com.au/ | National | Seniors (car insurance customers) | - Licensing of older drivers (including conditional licences and reporting medical conditions)  - Guidance on when an elderly family member or friend may be unfit to drive, and how to have that conversation | - Search bar - Referenced articles - Internal links | - Includes **references** to peer-reviewed journal articles, which are not commonly found on other senior driving websites |
| Australian Seniors | Government sources, statistics, direct quotes from experts, organisations like the Australian Road Safety Foundation. Some articles not updated regularly | https://www.seniors.com.au/ | National | Over 50s insurance | - Licence regulations by state - Advice on how to assess if a loved one is unsafe to drive - Information on **driving technologies** (e.g., ADAS) for seniors - Impact **of ageing on driving** (vision, hearing, reaction times, medications, etc.).  - Tips on **safe driving and vehicle maintenance** for seniors | - Search bar  - Blogs/news articles  - Shareable content  - FAQs  - Social media links  - Internal and external links  - Statistics  - Infographics  - Lists (e.g., top ten driving tips) | - **ADAS technologies:** Details on obtaining and using ADAS, including associated costs.  - Focus on maintaining **driving independence** while ensuring safety for seniors. |
| RACT | Not well-sourced, refers to Tasmanian government resources | https://www.ract.com.au/ | TAS | Insurance for Tasmanians | - Staying Mobile Hub for senior safety on the road - Road rules - Safe driving tips - Health and driving conditions - Tips for driving wellness - Prolonging fitness to drive - Vehicle safety features and assistive technologies - Driver training refreshers - Dementia and driving - Vehicle maintenance | - Search bar - Stock images - Quizzes (road rules, safe driving) - Video series (vehicle safety) - Articles - External and internal resource links - Brochures/fact sheets | - 'Hub' for senior safety on the road - Focus on 'driving wellness' (healthy driving) - Video series format instead of individual videos |
| Senior Driver Assessments | Not sourced, appears to use some information from governmental sources | https://seniordriverassessments.com.au/ | QLD | Provides senior driver assessments | - Legal requirements for elderly drivers in QLD - Driving requirements in other states - Driving fitness assessments and their benefits - How aging affects driving ability - Driving safety tips - Signs that an elderly parent is unsafe on the road | - Blog  - Lists/checklists | - Emphasis on the importance and benefits of driving fitness assessments for individuals and the community |

Observations:

- Many government websites focus on topics such as quitting driving, the legal obligations of individuals with medical conditions or impairments, alternative transportation, and guidance for those concerned about a loved one's driving.
- A majority of the resources (e.g., videos, fact sheets, posters) offer general driving safety information rather than being specifically tailored for older drivers.
- Websites frequently link to other external resources for further information, rather than consolidating all content on a single platform.

**Discussion guide – older adult drivers**

Purpose: To explore older adults’ experiences with driving as they age, their information-seeking behaviours, and feedback on the prototype Ageing Well on the Road website.

Discussion topics and sample prompts:

*1. Information Search*

- Have you previously looked for information about driving or licensing for older adults?
- Where did you look, and why?
- How do you decide if a website is trustworthy?
- What kind of information would you find most useful?

*2. Use of online resources*

- Would you use a website to find information about driving safety?
- What features or topics would make a website helpful for you?
- Would you share this information with your GP or family?

*3. Website feedback*

- What are your first impressions of the prototype website?
- Which sections or features stood out as most or least useful?
- How easy was it to navigate and understand?
- Would you recommend it to others?

*4. Closing reflections*

- Is there anything else you’d like to add about online resources for older drivers?

**Discussion guide – clinicians**

Purpose: To explore clinicians’ experiences with Fitness to Drive (FtD) assessments, identify resource and training needs, and gather feedback on the Ageing Well on the Road website for use in clinical practice.

Discussion topics and sample prompts:

*1. Clinical information needs*

- What tools, resources, or training would help you feel more confident conducting FtD assessments?
- What online or in-practice materials do you currently use?
- How could training or resources be improved?

*2. Use of screening tools*

- Which screening tools do you use and why?
- How do you decide which tools are valid or appropriate?
- How do patients usually respond to these assessments?

*3. Current practices*

- When and how do FtD assessments typically occur in your practice?
- What challenges do you face when conducting them?
- What recommendations or referrals do you provide afterwards?

*4. Evaluating online resources*

- Do you use online searches for FtD guidance?
- How do you judge whether a website or resource is credible?
- What features (e.g. videos, case examples, FAQs) would make an online resource more useful?

*5. Website feedback*

- What are your first impressions of the *Ageing Well on the Road* website?
- Which sections or features are most or least relevant?
- Would you use or recommend it in your practice?
- What content or features could improve its usefulness for clinicians?

*6. Closing reflections*

- Any other thoughts about improving FtD resources or supporting older drivers in clinical settings?

**Coding framework – older adult drivers**

The coding framework reflects how data segments were grouped into initial codes, subthemes, and overarching themes. Codes relating to website feedback were developed with reference to the interview guide, which was informed by CFIR and TAM (e.g., perceptions of innovation, access to information, and usefulness). Codes relating to participants’ broader beliefs, information-seeking behaviours, and driving practices were generated inductively. The tables below illustrate examples of how codes were organised into subthemes and themes. Participant quotes are provided to demonstrate the analytic process. They are illustrative only and do not represent the full dataset.

| Broader Theme | Subtheme | Codes | Participant Quotes |
| --- | --- | --- | --- |
| Barriers to accessing information and resources | Reluctance to acknowledge driving limitations | Hesitation to recognise own driving issues, resistance amongst long-time drivers | “The biggest problem all around is persuading people… to use these things [online resources and tools]… persuading someone who isn’t really aware of the difficulties they are having” (P6) |
|  | Reactive information-seeking | Information sought only when prompted by personal or external cues | “You’re not going to be prompted to go looking for information that you don’t need at the moment” (P3) |
|  | Scepticism towards online resources | Concerns about accuracy, distrust of assessments, privacy fears | “I’m sceptical that a computer questionnaire can actually make an assessment on my ability to drive” (P4) |
|  | Complexity of licensing regulations | Confusion over state differences, unclear rules | “The laws are different in every state” (P7) |
| Facilitators of online resource use | Accessibility and convenience | Easy to access, time-saving, quick | “It’s very quick. It’s very easy. It’s very comprehensive” (P4) |
|  | Reducing anxiety and stigma | Non-judgemental information, less stressful than GP visits | “Every time I go to the GP, my blood pressure goes up… Outside the GP’s office my blood pressure is normal” (P5) |
|  | Advisory guidance and reassurance | Guidance rather than prescriptive outcomes, supportive feedback | “I wouldn’t mind as long as it’s a tool that’s helping you to determine if you’ve got some issues or not. But it’s not a definitive yes or no” (P1) |
| Driving safety and self-regulation | Proactive approaches | Seeking assessments, maintaining driving confidence | “My specialist suggested I have this occupational therapy driving assessment… I prepared for and did all the reading before that” (P2) |
|  | Social and family influence | Peer discussions, family concerns influence decisions | “Drivers will always talk to another driver about their ability or a problem” (P4)  “You’ll get feedback from others about driving” (P4) |
|  | Self-imposed driving restrictions | Voluntary limitation of driving, avoidance of difficult conditions | “My wife says that she isn’t going to drive after dark… She simply just said, ‘I don’t really feel that great driving after dark’” (P6) |
|  | Access to alternative transport | Urban vs rural differences, public transport availability | “People in big cities… often give up [driving] quite easily as they are near the things they want and the public transport isn’t too bad and it’s probably free… But country districts… it’s going to be important and difficult to deal with” (P6) |
| Motivations and resource needs for information search | Health-related prompts | Medical diagnoses trigger information-seeking | “I’ve got mild cognitive impairment… my specialist suggested I complete an occupational therapy assessment” (P2) |
|  | Family influence | Relatives’ health or experiences prompt searches | “My [relative] was affected by dementia, and she was very resistant to giving up the freedom of driving. I looked online… and just Googled generally about the issues” (P3) |
|  | Preferred sources of information | Trustworthy, credible, relevant, location-specific | “I wouldn’t look at anything American for example, so relevant to my location… it’s got to be a reputable source” (P7) |
|  | External prompts | Legal notices, licensing requirements, official reminders | “I got a letter in the mail saying you are due for a check-up” (P6)  “People in general don’t go looking for this stuff. It’s a push technique, not a pull” (P6) |
| Preliminary impressions of website | Usability | Clear, accessible, easy to navigate | “It wasn’t too detailed, and it was in plain English… easy to read” (P1) |
|  | Credibility | University affiliation, trustworthy content | “It’s got the University symbol… so that gives it some credibility” (P7) |
|  | Content elements | State-based licensing info, visual resources | “One aspect I found very good was that you had each State and Territory listed, and their licensing requirements” (P4) |

**Coding framework – General Practitioners**

The coding framework reflects how data segments were grouped into initial codes, subthemes, and overarching themes. Codes relating to website feedback were developed with reference to the interview guide, which was informed by CFIR and TAM (e.g., perceptions of innovation, access to information, and usefulness). Codes relating to participants’ broader beliefs, information-seeking behaviours, clinical workflow, and driving-related decision-making were generated inductively.

The tables below illustrate examples of how codes were organised into subthemes and themes. Participant quotes are provided to demonstrate the analytic process. They are illustrative only and do not represent the full dataset.

| Broader Theme | Subtheme | Codes | Participant Quotes |
| --- | --- | --- | --- |
| Barriers to accessing information and resources | Reluctance to acknowledge driving limitations | Limited patient insight, dependence on family to raise concerns, Delay in seeking advice or assessment | “It’s pretty rare… when a patient says ‘maybe I shouldn’t be driving’. I think that’s happened to me once. It may be family members that may say something, but it’s rare for the individual to say ‘no, I’m not good enough’.” |
| Facilitators of online use | Reducing anxiety and stigma | Fear of losing licence, catastrophising about outcomes, need for reassurance and neutral framing | “They get very anxious. Very worried they will fail and their licence will be taken away from them, and there’s a rapid ball of catastrophising that goes on.” |
| Driving safety and self-regulation | Self-imposed driving restrictions | Self-awareness of declining ability, adjusting driving habits voluntarily, preference for gradual change | “I find I have patients who self-restrict their own driving, and say, I no longer make big road trips… I only drive where I feel comfortable now…. they’ve just personally downgraded their driving status within their comfort level.” |
| Motivations and resource needs for information search | Health-related prompts and regulatory triggers | New diagnoses prompts, reliance on GP guidance | “Someone newly diagnosed as a diabetic… or a syncope episode or a stroke, those sorts of things” |
| Website feedback | Perceived clinical usefulness | Positive overall impression, potential for patient referral, desire for cost and evidence information | “Looks great… I’d absolutely refer patients to this.”  “Suggestion to add some statistics and evidence to the website”  “I find one of the big things is patients saying how it’s expensive to use taxis, but when you point out to them that you have the sale of the vehicle, plus registration fees and fuel fees, they would have quite a lot of taxi/uber money banked. That financial side of things would be useful to have in there” |

**Mapping of User Feedback to Website Refinements**

This table outlines how feedback from older drivers, general practitioners, and stakeholders directly informed refinements to the Ageing Well on the Road website before its public release. This does not represent all the data collated.

| Collated Feedback | Website Section Affected | Action Taken Before Launch |
| --- | --- | --- |
| Front page not “enticing” enough | Home page | Simplified layout, added a concise introduction, and clearer text |
| Difficulty navigating between sections, too much scrolling | All pages | Added site-wide navigation bar, added “Back to Top” button after each section for easier navigation |
| Some images showed left-hand drivers or younger drivers (not relevant to target audience) | Multiple pages (Vision, Medication, Skills, Technology, Retiring from Driving) | Replaced images to ensure right-hand drivers and older adult representation |
| Roundabout and Give Way instructions were confusing | Knowledge & Skills – Road Rules | Reworded phrasing and clarified confusing wording |
| Include cautionary advice about medications affecting driving | Health & Driving – Medication section | Added more clinical information, and added disclaimer “If in doubt about the effects of medications on your ability to drive, continue medication but avoid driving until you have discussed this with your GP.” |
| Some links were broken and some linked to out-of-date resources | Licensing & Retiring from Driving | Updated all links to current State and Territory handbooks (e.g., COTA ACT 2023) |
| Need for credibility cues and research-based evidence | Home page & About Us | Added UNSW and ACT Road Safety Fund acknowledgements, cited key studies, and best evidence supporting advice to increase the credibility of the website |
| Suggestion to separate information for family/friends, tailored to each audience | Retiring from Driving | Created distinct “For Family and Friends” subsections with communication guidance and links for both audiences, as information differs |
| Desire for more visuals and accessible formatting | Skills, Heavy Vehicles, Technology pages | Added more infographics and diagrams (e.g., heavy vehicle blind spot image), added Accessibility tool plug-in to website |
| Request for consistent States and Territory page organisation and tile order | Technology in Cars, Licensing | Reorganised tiles to follow logical order and be consistent throughout the entire website |
| Update language “accident” → “crash” | Technology in Cars & multiple pages | Revised terminology site-wide to “crash” as confirmed by experts in the field |
| Include links to validated screening tools | For Clinicians | Updated tool links, added a clear section on validated screening tools |
| Include additional support information | All pages | Added video content from an occupational therapist explaining key concepts and management strategies throughout the entire website |
